# Clinical and Metabolic Effects of Incretin-Based Pharmacotherapy in Normal-Weight Patients With Type 2 Diabetes: A Real-World Analysis

**DOI:** 10.7759/cureus.88898

**Published:** 2025-07-28

**Authors:** Erin Carson, Fatima Khaleq, Cassidy McDonald, Nadine Smith, Alanis Rodriguez Colon

**Affiliations:** 1 Pharmacy Practice, University of Illinois at Chicago, Rockford, USA; 2 Pharmacy Department, University of Wisconsin, Madison, USA

**Keywords:** diabetes type 2, glp1-ra, glucagon-like peptide-1 receptor agonists, hemoglobin a1c (hba1c), normal weight, weight reduction

## Abstract

Purpose

To evaluate the impact of incretin-based pharmacotherapy on clinical outcomes in normal to underweight patients with type 2 diabetes mellitus in a real-world setting.

Methods

Patients with a body mass index less than 25 kg/m^2^ prescribed a glucagon-like peptide-1 receptor agonist or glucagon-like peptide-1 receptor agonist/glucose-dependent insulinotropic polypeptide receptor agonist for type 2 diabetes mellitus within a single health system were included in this analysis. The primary objective is a change in hemoglobin A1c from baseline to at least three months after an optimally tolerated incretin-based pharmacotherapeutic agent dose. Key secondary objectives include the absolute change in weight, low-density lipoprotein (LDL) levels, and systolic blood pressure from baseline to optimization, as well as the absolute change in the number of diabetes, hypertension, and cholesterol medications. Additionally, the number of patients who meet the categorization as underweight from baseline to optimization will be assessed.

Results

Within the health system described, 100 patients out of the 7942 prescribed an incretin-based pharmacotherapeutic agent within the two-year timeframe met inclusion criteria. Of these, 45 were on the medication for at least six months and had complete post-optimization records and so were included in the analysis. Within this population, the average baseline hemoglobin A1c was 8.83%, and after dose optimization, it was 7.98%. The average hemoglobin A1c change was -0.84% (P = 0.008) from baseline to optimization. The average baseline weight in the population analyzed was 64.3 kg, and after dose optimization, it was 62.7 kg. The average change in weight after optimization was -1.61 kg (P = 0.01), and the average change in body mass index after optimization was -0.45 kg/m^2^ (P = 0.38). The average changes in LDL and systolic blood pressure after optimization were -10 mg/dL (P = 0.02) and -4 mmHg (P = 0.28), respectively.

Conclusion

An observation of significantly improved hemoglobin A1c was seen in this real-world analysis of normal-weight patients with type 2 diabetes mellitus prescribed incretin-based pharmacotherapy. Weight loss was modest, and the change in body mass index was not statistically significant. These findings suggest that incretin-based pharmacotherapy is effective in improving glycemic control in this population with modest weight reduction. Further studies are needed to explore the long-term efficacy and safety of incretin-based pharmacotherapy in normal and underweight individuals with type 2 diabetes mellitus.

## Introduction

As new medications continue to emerge for the treatment of diabetes, healthcare professionals face an increasing array of questions regarding the latest guidelines and best practice recommendations. This evolving landscape highlights the importance of understanding how these guidelines address diverse populations, particularly underrepresented groups who may have unique health challenges and treatment responses. Ensuring that evidence-based recommendations are inclusive and applicable to all demographics is crucial for optimizing diabetes care and reducing health disparities. As we navigate these complexities, it is essential to critically evaluate how new therapeutic options can be effectively integrated into clinical practice to meet the needs of all patients. The inclusion of diverse populations in clinical studies is vital for ensuring that research findings are relevant and applicable to a wide range of individuals. Unfortunately, certain groups, particularly those categorized by weight status, age, gender, and ethnicity, are frequently excluded or underrepresented in clinical trials [1]. This issue prompted the creation of the National Institutes of Health (NIH) Revitalization Act in 1993, aimed at increasing diversity in clinical research and reducing healthcare disparities. Addressing these disparities is essential for advancing health equity and ensuring that clinical research accurately reflects the populations it seeks to serve.

According to the Centers for Disease Control and Prevention (CDC), about 90% of patients with type 2 diabetes mellitus (T2DM) are classified as overweight or obese [2]. This statistic highlights a significant oversight in clinical research: a substantial number of normal-weight and underweight patients are often excluded from studies evaluating medication treatments for this disease state. This lack of representation raises important questions about the effectiveness and safety of these medications for the full spectrum of T2DM patients.

One such class of medications that has shown considerable promise in treating diabetes is incretin-based pharmacotherapies, including glucagon-like peptide-1 receptor agonists, or GLP-1 RAs, plus or minus glucose-dependent insulinotropic polypeptide receptor agonists (GLP-1/GIP RAs). These medications, which include but are not limited to liraglutide, dulaglutide, semaglutide, and tirzepatide, are incredibly effective for the treatment of diabetes [3-12]. Tirzepatide is the only medication within this class with the additional GIP RA mechanism, so for the sake of terminology consistency throughout this article, all medications within this class, including tirzepatide, will be referred to under the term “incretin-based pharmacotherapy” except when it is pertinent to highlight tirzepatide’s additional mechanism. This class has been shown to lower hemoglobin A1c (HbA1c) by 1-2.1%, depending on the agent [3-12]. However, they are also known to cause an average weight loss of 9-20% over the course of 56 to 72 weeks, again depending on the agent [3-12]. This is generally beneficial, considering that 90% of patients with type 2 diabetes are overweight or obese [2]. However, it is important to consider the effects of an agent that promotes such weight loss on patients who are of normal weight or underweight. Normal-weight and underweight patients were excluded from the tirzepatide phase 3 clinical trials [3,4]. While not excluded from phase 3 clinical trials of other GLP-1 RAs, the reported mean BMI in these trials (30.3-33.9 kg/m^2^) suggests underrepresentation [5-12]. This underrepresentation may lead to inappropriate dosing recommendations and increased risks of adverse effects, as treatments may not be adequately tested for individuals with different metabolic profiles [1]. As a result, healthcare providers may face challenges in delivering evidence-based care tailored to the needs of normal and underweight patients, further exacerbating health disparities and compromising overall patient outcomes.

Beyond phase 3 trials, the evidence supporting or refuting the use of these agents in this underrepresented population is disappointingly sparse. While the American Diabetes Association Guidelines recommend incretin-based pharmacotherapy for patients with type 2 diabetes irrespective of baseline weight, only four studies examining GLP-1 RA and/or GLP-1/GIP RA efficacy in patients of normal weight or underweight in any capacity were identified. In these studies, most of the population is still overweight or obese, with only a minority of patients representing the underrepresented group [13-17]. Additionally, none of these studies examine semaglutide or tirzepatide, the two medications within this class shown to have the biggest impact on weight [3-12]. Despite this limited evidence, clinical guidelines do still recommend these agents, and they are being used in this population [13].

The objective of this retrospective review is to evaluate the impact of incretin-based pharmacotherapy on clinical outcomes in normal to underweight patients with type 2 diabetes mellitus (T2DM) in a real-world setting. Specifically, the analysis aims to assess changes in key clinical and metabolic parameters, including HbA1c levels, weight, blood pressure, and cholesterol levels from baseline to at least three months after optimally tolerated incretin-based pharmacotherapy. Additionally, this analysis evaluates the potential change in the number of daily medications required for the management of these conditions. By focusing on this underrepresented population, the analysis seeks to provide valuable insights into the effectiveness of incretin-based pharmacotherapy, ultimately contributing to improved healthcare strategies for individuals who may respond differently to diabetes management therapies.

## Materials and methods

All patients aged 18-75 prescribed a GLP-1 RA and/or GLP-1 RA/GIP RA for the treatment of T2DM within a single health system from January 1, 2023, through December 31, 2024, were included in this analysis. This timeframe was selected to ensure inclusion of tirzepatide within the data set, as this was the last of the included drugs to gain FDA approval. Patients were excluded for an indication other than T2DM, pregnancy, and a baseline BMI ≥25 kg/m^2^. For the purposes of this analysis, the baseline was considered to be the most recent measurement recorded in the medical record prior to initiation of the index medication. Additionally, patients with incomplete medical records or those who did not complete at least six consecutive months of incretin-based pharmacotherapy were excluded from the analysis. 

A retrospective chart review was performed in patients who completed at least six consecutive months of incretin-based pharmacotherapy to identify changes in key clinical and metabolic parameters, including HbA1c levels, weight, blood pressure, and cholesterol levels. Changes in the number of medications prescribed for diabetes, hypertension, and hyperlipidemia before and after starting incretin-based pharmacotherapy were also recorded. If patients meeting inclusion criteria were prescribed incretin-based pharmacotherapy and stopped therapy prior to six months, the reasons for discontinuation were recorded. Objective data were generated via reports from the electronic medical record. Any subjective data was collected via at least two individuals separately reviewing the medical record and reaching consensus. The analysis was approved by the institutional review board at all pertinent institutions. A waiver of informed consent was granted, given the retrospective nature of this analysis. All data were collected confidentially.

The primary objective of the retrospective analysis was to determine the change in HbA1c from baseline to at least three months after the patient reached the optimal tolerated dose. This objective was selected to align as much as possible with the primary objectives of many of the phase 3 clinical trials studying GLP-1 RA and GLP-1 RA/GIP RA medications within the scope of this real-world retrospective analysis. Secondary objectives included absolute change in weight from baseline to optimization, absolute change in low-density lipoprotein (LDL) from baseline to optimization, absolute change in systolic blood pressure from baseline to optimization, percentage of patients after optimization with a systolic blood pressure <140 mmHg, percentage of patients after optimization with an HbA1c <8%, absolute change in number of diabetes, hypertension, and cholesterol medications from baseline to optimization, and number of patients who meet categorization as underweight from baseline to optimization.

Descriptive statistics were utilized to report on primary and secondary objectives. A paired, two-tailed t-test was used to report p-values. A per-protocol analysis was conducted, which included only patients who adhered to the analysis protocol, were on incretin-based pharmacotherapy for at least six consecutive months, and had an HbA1c measurement recorded at least three months after reaching the maximum tolerated dose. Patients who did not have an HbA1c measurement after reaching the maximum tolerated dose were excluded from the analysis. The real-world results from this single-center retrospective review were compared to those published in phase 3 clinical trials of GLP-1 RAs and GLP-1 RA/GIP RAs to evaluate how the findings in this cohort of normal-weight and underweight patients align with the outcomes reported in larger populations. Secondary endpoints were similarly evaluated.

## Results

**Figure 1:**
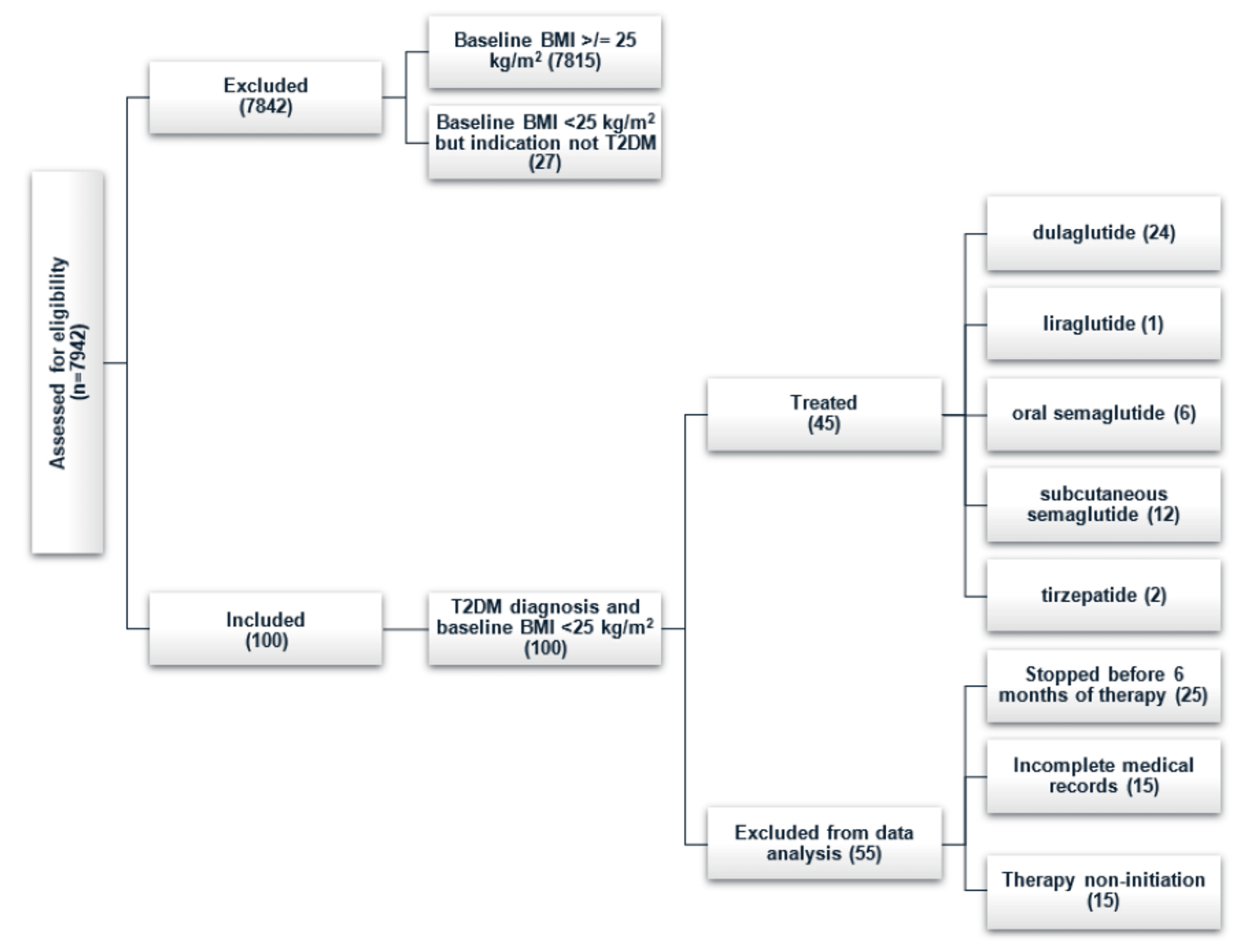
Patient disposition. T2DM: type 2 diabetes mellitus.

Within the single health system described in this analysis, GLP-1 RA and GLP-1 RA/GIP RA prescriptions were generated for 7942 unique patients between January 1, 2023, and December 31, 2024. Of these, 100 were patients with a diagnosis of T2DM and a baseline BMI of <25 kg/m^2^, as detailed in Figure 1.

Of those, 15 never started the medication prescribed, and 25 stopped prior to six months of therapy. Ten additional patients stopped after more than six months of therapy but before post-dose optimization data could be collected and were thus excluded from data analysis due to incomplete medical records. A further five patients were excluded from the final analysis due to not having a final HbA1c measurement after reaching the maximum tolerated dose.

Of the 45 patients included in this analysis, the average age was 58, the average baseline BMI was 23.15 kg/m^2^, and 27 (60%) were female, as described in Table 1. The specific initial incretin-based agents prescribed were dulaglutide (24), liraglutide (1), oral semaglutide (6), subcutaneous semaglutide (12), and tirzepatide (2). The optimally tolerated dose was the highest FDA-approved dose of the medication prescribed for 11 patients on dulaglutide, one patient on liraglutide, four patients on oral semaglutide, and six patients on subcutaneous semaglutide. All other patients were on therapeutic doses of these medications but stopped titration below the highest FDA-approved dose for various reasons related to both tolerability and blood glucose-lowering effectiveness. Thirteen patients required an in-class medication switch during the timeframe of interest, primarily due to not meeting clinical goals (eight patients, 61.5%) or due to backorder and access issues (five patients, 38.5%).

**Table 1:** Baseline characteristics. LDL: low-density lipoprotein.

Baseline characteristics	
Average age	58
Female %	60%
Average baseline weight	64.3 kg (range: 48.9 kg-88.2 kg)
Average baseline BMI	23.15 kg/m^2^ (range: 19.08 kg/m^2^-24.96 kg/m^2^)
Underweight at baseline %	0%
Average baseline A1c	8.83% (range: 5.5%-13.1%)
Average baseline LDL	105 mg/dL (range: 55 mg/dL-249 mg/dL)
Average baseline systolic blood pressure	127 mmHg (range: 100 mmHg – 171 mmHg)
Medications at baseline	
Number of anti-diabetic medications	2.4 (range 1-6)
% On at least one anti-diabetic medication	100%
Number of anti-hypertensive medications	1.1 (range 0-3)
% On at least one anti-hypertensive medication	73%
Number of cholesterol medications	0.9 (range 0-2)
% On at least one cholesterol medication	78%
Initial incretin-based agent prescribed	
Dulaglutide	24
Liraglutide	1
Oral semaglutide	6
Subcutaneous semaglutide	12
Tirzepatide	2

The primary objective of this analysis was to determine the change in HbA1c from baseline to at least three months after the patient reached the optimal tolerated medication dose. The average baseline HbA1c was 8.83%, with 13 patients (28.9%) having a baseline HbA1c <8%. The average HbA1c at least three months after dose optimization was 7.98%, with 27 patients (60%) having an HbA1c <8% after dose optimization, as described in Table 2. The average change in HbA1c was -0.84%.

**Table 2:** Effectiveness measures. LDL: low-density lipoprotein.

End points	Mean (range) at baseline, before starting incretin-based agent	Mean (range) at least three months after reaching optimal dose	Average change	Test statistic (t)	P-value
HbA1c, <8%	8.83% (5.5%-13.1%)	7.98% (5.5%-13.3%)	-0.84%	-2.76	0.008
Weight, kg	64.3 kg (48.9 kg-88.2 kg)	62.7 kg (43.5 kg-85.7 kg)	-1.61 kg	-2.60	0.01
Weight, BMI	23.15 kg/m^2^ (19.08 kg/m^2^-24.96 kg/m^2^)	22.58 kg/m^2^ (17.51 kg/m^2^-24.87 kg/m^2^)	-0.45 kg/m^2^	-1.99	0.38
LDL, mg/dL	105 mg/dL (55 mg/dL-249 mg/dL)	92 mg/dL (42 mg/dL-234 mg/dL)	-10 mg/dL	-2.45	0.02
Systolic blood pressure, <140 mmHg	127 mmHg (100 mmHg-171 mmHg)	123 mmHg (90 mmHg-170 mmHg)	-4 mmHg	-1.09	0.28
Number of anti-diabetic medications	2.4 (1-6)	2.7 (1-5)	-	-	-
Number of anti-hypertensive medications	1.1 (0-3)	0.9 (0-3)	-	-	-
Number of anti-hyperlipidemic medications	0.9 (0-2)	0.9 (0-2)	-	-	-
P-values were calculated using paired, two-tailed t-tests. Test statistic values (t) are reported in the second-to-last column.					

This analysis was also interested in describing the change in weight from baseline to optimization. The average baseline weight was 64.3 kg. The average baseline BMI was 23.15 kg/m^2^, with zero patients considered to be underweight based on a BMI of <18.5 kg/m^2^. The average weight after dose optimization was 62.7 kg. The average BMI after optimization was 22.58 kg/m^2^, with one patient (2%) considered to be underweight based on a BMI of <18.5 kg/m^2^. The average change in weight after optimization was -1.61 kg, and the average change in BMI after optimization was -0.45 kg/m^2^.

Other metabolic parameters described in this analysis include a change in LDL and a change in systolic blood pressure. The average baseline LDL was 105 mg/dL. The average LDL after dose optimization was 92 mg/dL. The average change in LDL after optimization was -10 mg/dL. The average baseline systolic blood pressure was 127 mmHg, with 26 patients (80%) having a systolic blood pressure <140 mmHg at baseline. The average systolic blood pressure after dose optimization was 123 mmHg, with 41 patients (91%) having a systolic blood pressure <140 mmHg after optimization. The average change in systolic blood pressure was -4 mmHg.

The number of anti-diabetic medications prescribed at baseline, not including the incretin-based agent, was 2.4. All patients (100%) were on at least one anti-diabetic medication at baseline. The number of anti-diabetic medications prescribed after optimization, including the incretin-based agent, was 2.7. Again, all patients (100%) were on at least one anti-diabetic medication after optimization. The number of antihypertensive medications prescribed at baseline was 1.1. Thirty-three patients (73%) were on at least one antihypertensive medication at baseline. The number of antihypertensive medications prescribed after optimization was 0.9. Twenty-eight patients (62%) were on at least one antihypertensive medication after optimization. The number of cholesterol medications prescribed at baseline was 0.9. Thirty-five patients (78%) were on at least one cholesterol medication at baseline. The number of cholesterol medications prescribed after optimization was 0.9. Again, 35 patients (78%) were on at least one cholesterol medication after optimization.

## Discussion

This analysis provides valuable real-world data on the effectiveness of incretin-based pharmacotherapy in patients with T2DM who are normal weight or underweight, a population that has often been underrepresented in clinical trials [3-12]. Despite the limitations inherent in retrospective analyses, such as the potential for selection bias, variability in titration protocols and baseline data timeframes, and the absence of a control group, the findings offer important insights into how incretin-based pharmacotherapy can affect key clinical outcomes in this demographic. This analysis highlights the need for further research to better understand how incretin-based pharmacotherapy impacts underrepresented patient groups and to develop more tailored treatment guidelines that ensure the safety and efficacy of these therapies for all individuals with T2DM.

While the inherent nature of this retrospective analysis precludes a control group, results from this cohort can be compared to those seen in other trials. Since HbA1c reduction is the primary outcome in major phase 3 trials studying these drugs, it is reasonable to compare this outcome when considering effectiveness in this cohort. In terms of HbA1c reduction, the data from this cohort suggest that the effectiveness of incretin-based pharmacotherapy aligns with the benefits observed in larger clinical trials, although the degree of improvement was somewhat less pronounced. The baseline HbA1c in this analysis, 8.83%, was comparable to the baseline HbA1c in several phase 3 trials, such as SURPASS-4, SUSTAIN-5, and AWARD-11, where participants had mean baseline HbA1c levels ranging from 8.3% to 8.6%, which may imply similarity in the degree of T2DM control between cohorts [4,6,10]. The HbA1c reduction observed in this cohort was statistically significant, consistent with the positive outcomes seen in phase 3 trials [3-12]. After dose optimization, the average HbA1c reduction in this cohort was -0.84% (P = 0.008), which is similar to reductions in the AWARD-2 and AWARD-3 trials for dulaglutide 1.5 mg (-1.08% and -0.78%, respectively) [8,9]. However, tirzepatide was prescribed to only two patients in this cohort, and tirzepatide phase 3 trials showed the greatest HbA1c reductions among incretin-based pharmacotherapy [3,4]. Additionally, only 22 patients (48.8%) in this cohort achieved the optimally tolerated dose, which is lower than reported in most phase 3 clinical trials [3-12]. While the results of this analysis align with general trends seen in phase 3 trials in terms of HbA1c lowering, despite baseline weight, the variability in patient responses and dosing strategies may explain differences in the magnitude of HbA1c reduction observed in this analysis versus larger, more standardized clinical trials. This analysis describes HbA1c changes by class rather than by individual drug, as described in phase 3 trials. Furthermore, variability in dosing regimens across phase 3 trials between drugs, as well as different time frames between dose optimization and HbA1c analysis, could contribute to these discrepancies.

While the primary outcome in phase 3 trials is HbA1c reduction, the secondary outcome of weight is of particular interest to the cohort described in this analysis. The average weight change from baseline to optimization in this cohort was a decrease of -1.61 kg (P = 0.01). Though statistically significant, this modest weight loss is less than what is typically observed in studies with overweight or obese populations. For instance, in the PIONEER trials, oral semaglutide showed a range of weight reductions, from -1.7 kg in the cohort receiving oral semaglutide 3 mg daily over 26 weeks in the PIONEER 1 trial to -4.4 kg in the cohort receiving oral semaglutide 14 mg daily in the PIONEER 4 trial [11,12]. Weight loss was similar to or significantly greater than in all other phase 3 clinical trials of the GLP-1 RAs and GLP-1 RA/GIP RAs used in this cohort [3-12]. In particular, the weight loss observed in the SURPASS trials with tirzepatide was the greatest, with reductions of -6.2 to -12.9 kg depending on the dose of tirzepatide over 40-52 weeks [3,4]. Typical phase 3 reductions may account for some of this difference. However, only two patients in the cohort described in this analysis were prescribed tirzepatide, and none reached the optimally tolerated dose, which limits direct comparison to these higher reductions. However, when compared, weight changes described in phase 3 trials of GLP-1 RAs and GLP-1 RA/GIP RAs as a whole, a less pronounced weight change was observed in this cohort that started at a lower baseline weight and normal BMI.

There are limitations to this analysis inherent in the study design described above. For one, metabolic changes are slow. While the timeframe for reporting weight loss differs between all the phase 3 trials, demonstrating a lack of standardization on optimal timeframe reporting, likely the timeframe described in this cohort, from baseline to optimization, is not long enough to truly understand implications related to weight changes. Additionally, as with any single-center retrospective analysis, there is the potential for selection bias. Reasons why some normal-weight or underweight patients were selected to start incretin-based pharmacotherapy, while others were not, are beyond the scope of this analysis but must be considered when considering generalizability. Factors such as provider preferences, patient requests, baseline degree of diabetes control, and history of weight changes could all have contributed to the population described in this analysis. One notable finding is that every single patient included in this analysis was on at least one anti-diabetic medication at baseline. Only 1.2% of the prescriptions for incretin-based pharmacotherapy generated by the health system described in this analysis over the timeframe of interest were for patients meeting the inclusion criteria of a diagnosis of T2DM and a baseline BMI of <25 kg/m^2^, despite the fact that the CDC reports about 10% of patients with T2DM are classified as normal weight or underweight [2]. Incretin-based pharmacotherapy may be prescribed for a wide variety of reasons beyond the indication of T2DM, so limited conclusions can be made from this discrepancy, but it does limit the available sample size to analyze. It is additionally noteworthy that incretin-based pharmacotherapy, such as semaglutide and tirzepatide, was less commonly prescribed in this cohort, possibly due to concerns about weight loss in normal-weight and underweight individuals. This underscores the need for more targeted clinical guidance regarding the use of these medications in this population, which is often overlooked in the literature. 

The lack of a control group complicates comparisons with major clinical trials. It remains unclear whether the less pronounced HbA1c benefits and weight loss overall in this cohort were due to differences in baseline BMI between this population and the populations of larger trials or the myriads of other factors that can affect HbA1c and weight. If a comparator group had been considered, it would be difficult to control for confounding variables given the retrospective nature of this analysis. Additionally, the quality of the data analyzed must be considered. Retrospective studies often face challenges related to incomplete or inaccurate real-world records. In this analysis, baseline data were defined as the last data point prior to the initiation of the index medication. The timeframe for baseline data measurements could have varied widely from patient to patient. The same is true for post-optimization data, though the protocol ensured data was collected at least three months after the patient reached the optimal tolerated dose. Missing data, another problem inherent to the retrospective review, were also addressed and controlled for, though this did result in 15 patients being excluded from analysis because of missing or incomplete data.

Despite these limitations, this analysis provides a thoughtful and controlled approach to describing real-world outcomes. Efforts were made to control for confounding variables by ensuring patients were on therapy for at least six months, recognizing that metabolic changes are gradual and that titration takes time. Baseline percentages of patients were reported to have achieved various metabolic goals (HbA1c, systolic blood pressure) to provide context for the relative control of these disease states within this population. Any patients with missing data were excluded from this analysis. 

## Conclusions

This analysis provides real-world insights into the effectiveness of incretin-based pharmacotherapy in patients with normal weight or those who are underweight, a population often excluded or underrepresented in major clinical trials. By incorporating findings from phase 3 trials of semaglutide, tirzepatide, liraglutide, and dulaglutide, it is possible to consider those results in the context of this cohort, although the absence of a comparator group and the inherent limitations of retrospective data make it difficult to draw definitive conclusions. These patients are often overlooked in clinical research, and there are currently no other studies addressing this gap in the literature. As a result, this underrepresented patient population continues to be a challenge for clinicians seeking to make informed decisions about the use of incretin-based pharmacotherapy in this specific group. The lack of clear guidance underscores the need for further exploration into whether these agents should be used in normal-weight or underweight individuals. Ultimately, the findings from this analysis aim to inform clinical practice and improve patient outcomes for those who have been historically neglected in clinical trials, offering valuable insights for clinicians facing the challenge of deciding whether these therapies are appropriate for this underrepresented patient group.
